# FilGAP, a GAP for Rac1, down-regulates invadopodia formation in breast cancer cells

**DOI:** 10.1247/csf.23032

**Published:** 2023-07-22

**Authors:** Koji Saito, Sakino Ozawa, Yosuke Chiba, Ruri Takahashi, Ryoya Ogomori, Kojiro Mukai, Tomohiko Taguchi, Hiroyasu Hatakeyama, Yasutaka Ohta

**Affiliations:** 1 Division of Cell Biology, Department of Biosciences, School of Science, Kitasato University, Kanagawa, Japan; 2 Laboratory of Organelle Pathophysiology, Department of Integrative Life Sciences, Graduate School of Life Sciences, Tohoku University, Sendai, Japan; 3 Department of Physiology, School of Medicine, Kitasato University, Kanagawa, Japan

**Keywords:** invadopodia, breast carcinoma, Rac1, FilGAP, PI(3,4)P_2_

## Abstract

Invadopodia are protrusive structures that mediate the extracellular matrix (ECM) degradation required for tumor invasion and metastasis. Rho small GTPases regulate invadopodia formation, but the molecular mechanisms of how Rho small GTPase activities are regulated at the invadopodia remain unclear. Here we have identified FilGAP, a GTPase-activating protein (GAP) for Rac1, as a negative regulator of invadopodia formation in tumor cells. Depletion of FilGAP in breast cancer cells increased ECM degradation and conversely, overexpression of FilGAP decreased it. FilGAP depletion promoted the formation of invadopodia with ECM degradation. In addition, FilGAP depletion and Rac1 overexpression increased the emergence of invadopodia induced by epidermal growth factor, whereas FilGAP overexpression suppressed it. Overexpression of GAP-deficient FilGAP mutant enhanced invadopodia emergence as well as FilGAP depletion. The pleckstrin-homology (PH) domain of FilGAP binds phosphatidylinositol 3,4-bisphosphate [PI(3,4)P_2_], which is distributed on membranes of the invadopodia. FilGAP localized to invadopodia in breast cancer cells on the ECM, but FilGAP mutant lacking PI(3,4)P_2_-binding showed low localization. Similarly, the decrease of PI(3,4)P_2_ production reduced the FilGAP localization. Our results suggest that FilGAP localizes to invadopodia through its PH domain binding to PI(3,4)P_2_ and down-regulates invadopodia formation by inactivating Rac1, inhibiting ECM degradation in invasive tumor cells.

## Introduction

Malignant tumor cells acquire abnormal motility and invade through various extracellular matrix (ECM) components including basement membrane and interstitial collagen networks. The migrating tumor cells leave a blood or lymphatic vessel and invade the surrounding tissue parenchyma during tumor metastasis. (Bravo-Cordero *et al.*, 2012; Yamaguchi, 2012). The first migratory process for tumor cell invasion is the formation of invadopodia, which are actin-rich protrusive structures that mediate the ECM degradation (Linder *et al.*, 2011; Murphy and Courtneidge, 2011). The formation of invadopodia can be divided into several stages (Artym *et al.*, 2006; Eddy *et al.*, 2017; Murphy and Courtneidge, 2011). In the first stage, invadopodia precursors are formed by the initial accumulation of core components containing filamentous actin (F-actin) and cortactin. The next stage is the emergence and stabilization of protrusively invadopodia structures, characterized by actin polymerization through many actin-related regulators. In the third stage, mature invadopodia promote ECM degradation by matrix metalloproteases such as MT1-MMP. Finally, invadopodia are disassembled by disruption of their core components.

The formation of invadopodia is regulated by Rho small GTPases such as Rac1, Cdc42, and RhoA through the reorganization of the actin cytoskeleton (Masi *et al.*, 2020; Rivier *et al.*, 2021; Spuul *et al.*, 2014). Rho small GTPases function as molecular switches by cycling between the GTP-bound active state and the GDP-bound inactive state in cells (Jaffe and Hall, 2005). This cycle is governed by two classes of regulatory proteins. Guanine nucleotide exchange factors (GEFs) activate Rho GTPases by loading GTP, whereas GTPase-activating proteins (GAPs) facilitate the inactivation of Rho small GTPases by stimulating their intrinsic GTPase activity (Schmidt and Hall, 2002; Tcherkezian and Lamarche-Vane, 2007; van Buul *et al.*, 2014). Regard of the regulation of invadopodia structure and function, the specific functions of several GEFs and GAPs have been reported (Rivier *et al.*, 2021). Because the precise regulation of Rho small GTPase activity by these proteins should be required for the formation of invadopodia, it will need to be further elucidated which regulatory proteins, especially GAPs, mediate invadopodia formation and how their functions are regulated.

FilGAP (also called ARHGAP24) is a Rac-specific GAP that regulates cell morphology and cell spreading (Morishita *et al.*, 2015; Nakamura, 2013; Ohta *et al.*, 2006; Yamada *et al.*, 2016). In breast cancer cells, FilGAP controls cell shape changes during migration and contributes to cell invasion through the ECM *in vitro* (Saito *et al.*, 2021; 2012; Uehara *et al.*, 2017). In *in vivo* study, the depletion of endogenous FliGAP in breast cancer cells reduced tumor cell extravasation (Saito *et al.*, 2012). Therefore, FilGAP is thought to function as a positive regulator of the invasion of certain types of breast cancer cells. However, the role of FilGAP in invadopodia formation is unclear. In this study, we found that in breast cancer cells, FilGAP localizes to invadopodia through its pleckstrin-homology (PH) domain binding to phosphatidylinositol 3,4-bisphosphate [PI(3,4)P_2_] on membranes and down-regulates invadopodia formation by inactivating Rac1. We here propose that FilGAP plays a role in the suppression of invadopodia formation and excessive ECM degradation in the first process of tumor cell invasion.

## Materials and Methods

### Plasmids

The pCMV5-HA vector encoding FilGAP constructs (R175A and R39C) were previously described (Kawaguchi *et al.*, 2014; Ohta *et al.*, 2006). The pEGFP-c1 vector or pIRES2-AcGFP (Clontech-Takara Bio USA, Mountain View, CA, USA) encoding FilGAP constructs (full length [WT] or R39C) were described previously (Kawaguchi *et al.*, 2014; Saito *et al.*, 2012). The CSII-EF-MCS vector containing mCherry-tagged FilGAP KD#1-resistant construct (mCherry-FilGAP KD^r^) was described previously (Iida *et al.*, 2016). The pEGFP-c1-FilGAP R175A plasmid was generated by introducing point mutations using the QuikChange site-directed mutagenesis kit (Stratagene-Agilent, Santa Clara, CA, USA) as described previously (Saito *et al.*, 2012). The human Rac1 coding sequence was polymerase chain reaction (PCR)-amplified using a Rac1 construct, and the PCR products digested with EcoRI and XhoI were inserted into the pEGFP-c1 vector using the EcoRI and SalI sites and sequenced. The pcDNA3 vector (Invitrogen-Thermo Fisher Scientific, Waltham, MA, USA) encoding mCherry-tagged cortactin was generated as described previously (Saito *et al.*, 2021). The human cortactin cording sequence was PCR-amplified using a cortactin construct, and the PCR products digested with BamHI and EcoRI were inserted into the pcDNA3-mCherry plasmid using the BamHI and EcoRI sites and sequenced. NES-EGFP-cPHx3 (human PLEKHA1) plasmid (GFP-PLEKHA1) was purchased from Addgene (plasmid ID 116855, Cambridge, MA, USA).

### Antibodies and reagents

Mouse anti-α-tubulin (B-5-1-2), anti-cortactin (4F11), and anti-fish (TKS5, G-7) monoclonal antibodies were purchased from Sigma-Merck (Darmstadt, Germany), Millipore-Merck (Darmstadt, Germany), and Santa Cruz Biotechnology (Dallas TX, USA), respectively. Rabbit anti-HA and anti-SHIP2 polyclonal antibodies were purchased from Sigma-Merck and Cell Signaling (Danvers, MA, USA), respectively. Goat anti-GFP polyclonal antibody was purchased from Rockland immunochemicals (Gilbertsville, PA, USA). Rabbit anti-FilGAP polyclonal antibody was prepared as described previously (Ohta *et al.*, 2006). Secondary antibodies conjugated to Alexa Fluor 488, 568, or 647, Alexa Fluor 488 and 568-phalloidin (Invitrogen-Thermo Fisher Scientific), Oregon Green 488-gelatin (Invitrogen-Thermo Fisher Scientific), Hoechst33258 (Dojido Laboratories, Kumamoto, Japan), SHIP2 inhibitor (AS1949490, Sigma-Merck), Rac1 inhibitor (NSC23766, Merck), and epidermal growth factor (EGF, R&D Systems, Minneapolis, MN, USA) were also purchased from commercial sources. small interfering RNA (siRNA) oligonucleotide duplexes targeting human (NM_001025616) and rat (NM_001012032) *FilGAP* and rat *SHIP2* (NM_022944.2) were purchased from Invitrogen-Thermo Fisher Scientific. The targeting sequences were as follows: The targeting sequences were as follows: human *FilGAP* KD#1 5'-AAGAUAGAGUAUGAGUCCAGGAUAA-3' (nt 1975–1999) (Saito *et al.*, 2012), human *FilGAP* KD#2 5'-CAGUGAUGAUUAGCAAACAUGAUUG-3' (nt 956–980), rat *FilGAP* KD#3 5'-AAGTCACCATGGGTCAGTTACAGAA-3' (nt 1055–1079), rat *SHIP2* KD#1 5'-GAACCTGACATGATCTCCGTCTTCA-3' (nt 1261–1285), and rat *SHIP2* KD#2 5'-CCTTCATGTTCAATGGCACTTCTTT-3' (nt 1643–1667). Human *FilGAP* siRNA KD#1 has a single-base mismatch to the rat *FilGAP* mRNA (underline), but significantly reduced the expression of endogenous FilGAP in rat MTLn3 cells (see Fig. 4C). Negative control siRNA was also purchased from Invitrogen-Thermo Fisher Scientific (#452001).

### Cell culture and transfection

Human breast carcinoma cell line MDA-MB-231 and rat mammary adenocarcinoma cell line MTLn3 were cultured as previously described (Donnelly *et al.*, 2017; Saito *et al.*, 2021). Cells were transfected with plasmid DNA for 24 hours or siRNA for 48 hours using Lipofectamine 2000 (Invitrogen-Thermo Fisher Scientific) according to the manufacturer’s instructions. For co-transfection of plasmid DNA and siRNA, cells were first transfected with siRNA for 24 hours and then co-transfected with plasmid DNA, followed by additional culture for 24 hours. For a stable cell line of MDA-MB-231 cells overexpressing FilGAP, cells were transfected with the pIRES2-AcGFP vector (control) or pIRES2-AcGFP encoding *FilGAP* and selected at ~1 mg/ml geneticin (G418) (Invitrogen-Thermo Fisher Scientific). For siRNA rescue experiments, MDA-MB-231 cells were transfected by lentivirus infection with CSII-EF-MCS vector encoding *mCherry-FilGAP* KD^r^. Cell culture on gelatin was performed as previously described (Martin *et al.*, 2012). Gelatin was coated on glass coverslips (Matsunami Glass, Osaka, Japan) for immunofluorescence or glass bottom dishes (MatTek, Ashland, MA, USA) for time-lapse imaging.

### Immunoblotting

To check the efficiency of FilGAP depletion by siRNAs and the expression level of FilGAP in stable cell lines, the cells were solubilized with 1% sodium dodecyl sulfate (SDS) in phosphate-buffered saline (PBS) containing protease inhibitor cocktail (Sigma-Merck) and 1 mM phenylmethylsulfonyl fluoride (PMSF). Cell lysates were separated by SDS-PAGE and immunoblotting was performed as previously described (Saito *et al.*, 2021).

### Immunofluorescence and microscopic observation

**Fig. 1:**
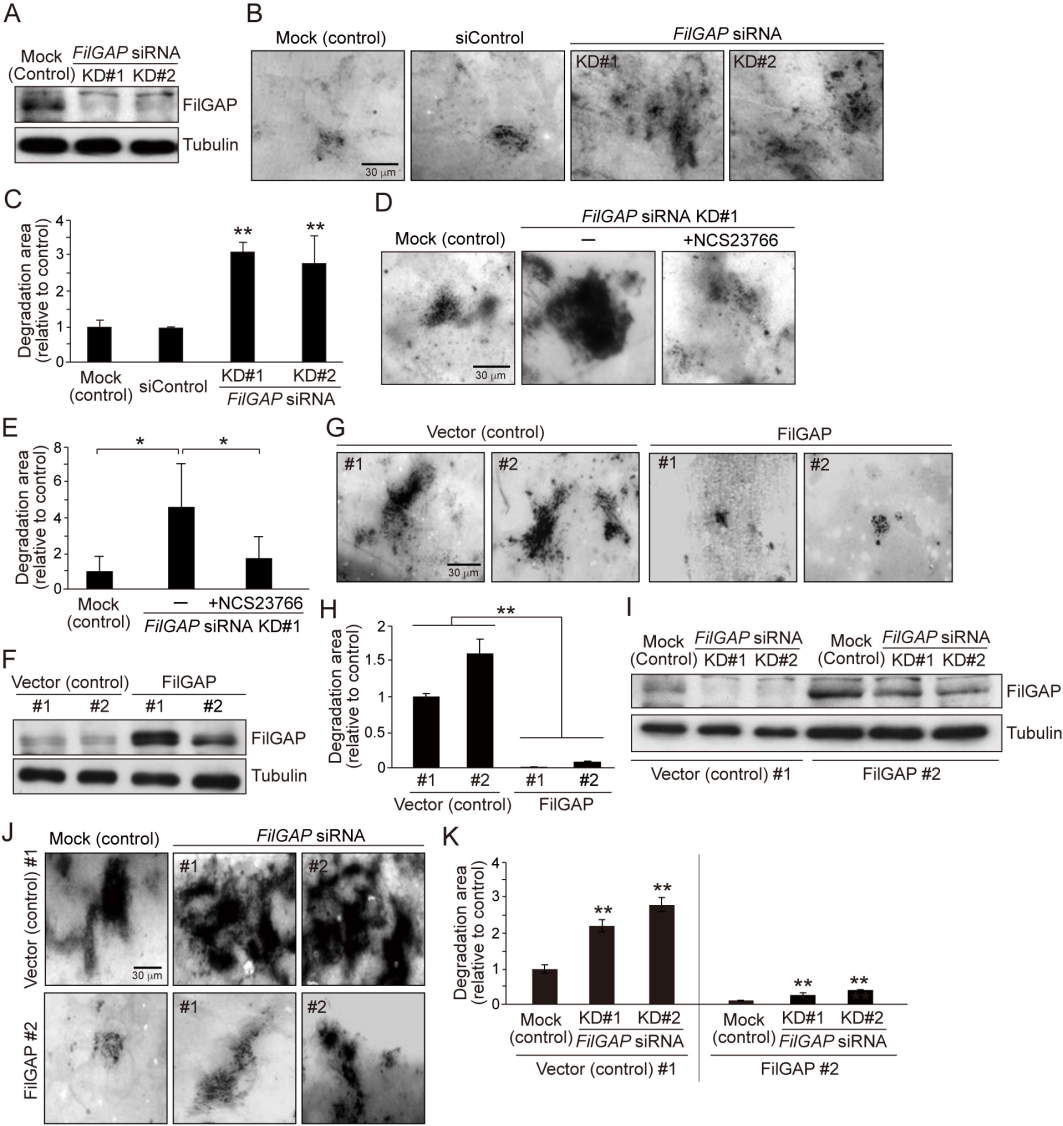
FilGAP inhibits ECM degradation of MDA-MB-231 cells (A) Immunoblot showing that FilGAP was depleted by *FilGAP* siRNAs in MDA-MB-231 cells. FilGAP and tubulin (loading control) were detected by immunoblotting using anti-FilGAP and anti-tubulin antibodies, respectively. (B) Images of gelatin degradation in no siRNA-transfected (Mock), negative control siRNA (siControl)-transfected, and FilGAP-depleted MDA-MB-231 cells. MDA-MB-231 cells were seeded on fluorescent gelatin and cultured for 24 hours for degradation. After the culture, the cells were fixed and stained with phalloidin for F-actin to visualize the cell area. (C) Quantification of the relative degradation area of the cells shown in B (see Materials and Methods). Statistical significance was determined by one-way ANOVA and Dunnett multiple comparison test (vs mock cells, N = 3, **p<0.01). (D) Images of gelatin degradation in FilGAP-depleted MDA-MB-231 cells treated with Rac1 inhibitor. The cells seeded on the gelatin were treated with 100 μM Rac1 inhibitor (NSC23766) for 24 hours. (E) Quantification of the relative degradation area of the cells shown in D. Statistical significance was determined by one-way ANOVA and Tukey multiple comparison test (N = 3, *p<0.05). (F) Generation of MDA-MB-231 cells stably overexpressing FilGAP (see Materials and Methods). Two stable cell lines (#1 and #2) were established and expression of FilGAP was confirmed by immunoblotting. The vector-transfected cell lines (#1 and #2) were also established as controls. (G) Images of gelatin degradation in control and FilGAP-overexpressing MDA-MB-231 cells shown in F. (H) Quantification of the relative degradation area of the cells shown in G. Statistical significance was determined as in E (N = 3, **p<0.01). (I) Depletion of FilGAP in the stable cell lines (control #1 and FilGAP #2) shown in F. (J) Images of gelatin degradation in control and FilGAP-depleted cells shown in I. (K) Quantification of the relative degradation area of the cells shown in J. Statistical significance was determined as in C (vs mock cells, N = 3, **p<0.01).

**Fig. 2:**
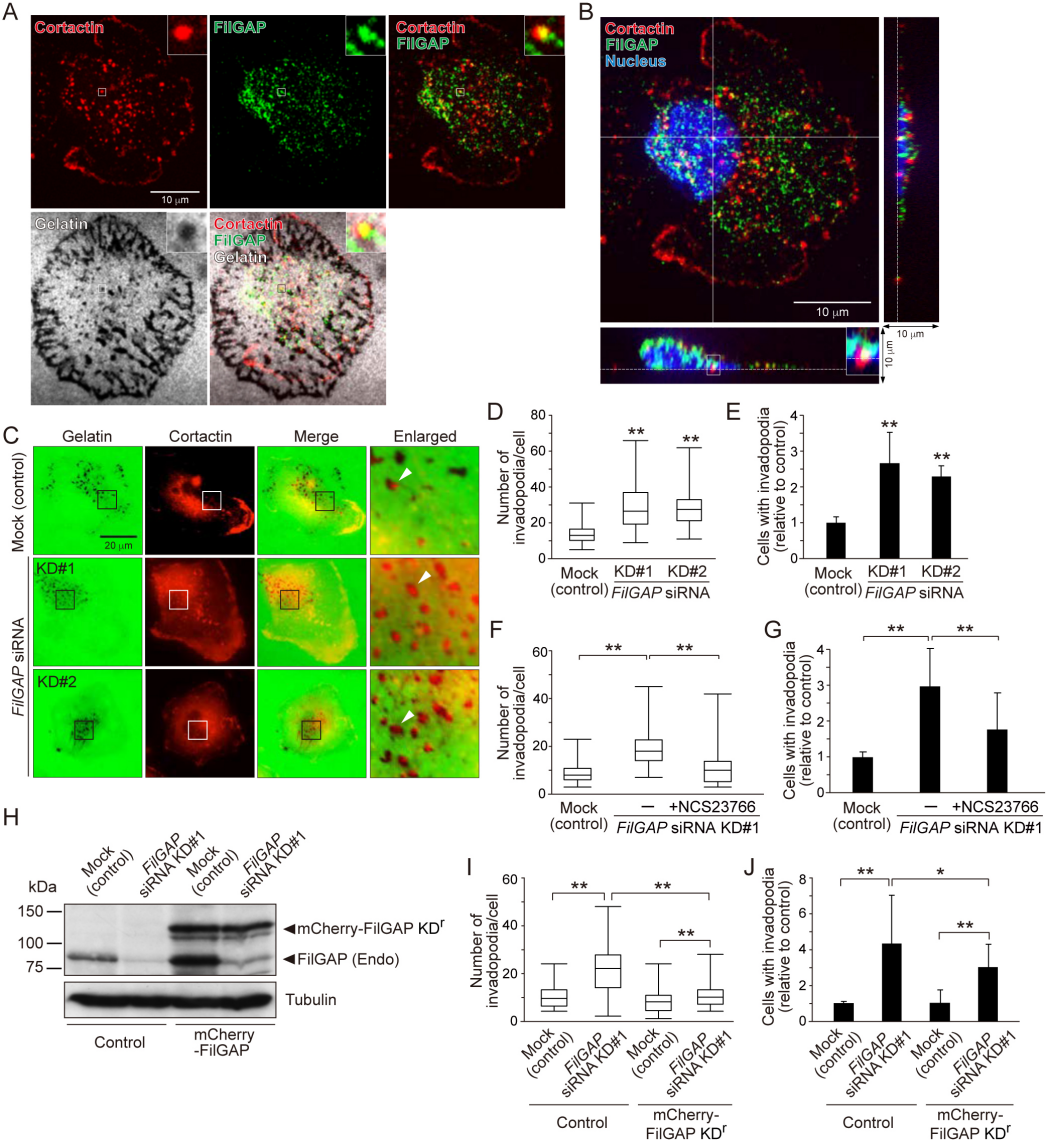
FilGAP localizes to invadopodia and depletion of FilGAP promotes invadopodia formation in MDA-MB-231 cells (A) Localization of FilGAP at the invadopodia in MDA-MB-231 cells on the gelatin. MDA-MB-231 cells were seeded on the fluorescent gelatin and cultured for 6 hours. The cells were fixed after CSK treatment (see Materials and Methods) and stained with anti-FilGAP and anti-cortactin antibodies. Insets show enlarged images of the boxed regions. Merge images are also shown. (B) Confocal z-stack image of the cell shown in A. The nucleus was visualized by Hoechst staining. Dotted lines in z-axis images indicate the ventral surface of the cells on the gelatin. Inset in the z-axis image shows an enlarged image of the boxed region. (C) Images of invadopodia with ECM degradation in control and FilGAP-depleted MDA-MB-231 cells. MDA-MB-231 cells were seeded on fluorescent gelatin and cultured for 6 hours. After the culture, the cells were stained with an anti-cortactin antibody. Merged and enlarged images of the boxed regions are shown. Arrowheads indicate representative invadopodia with gelatin degradation and cortactin staining, and the dots were analyzed in D and E. (D) Quantification of the number of invadopodia per cell of the cells shown in C (see Materials and Methods). Box and whisker plots indicate median, quartiles, and highest and lowest values. Statistical significance was determined by one-way ANOVA and Dunnett multiple comparison test (vs mock cells, n = 30 cells, **p<0.01). (E) Quantification of the relative number of cells with invadopodia shown in C. Statistical significance was determined as in D (vs mock cells, N = 3, **p<0.01). (F) and (G) Quantification of FilGAP-depleted MDA-MB-231 cells treated with 100 μM NSC23766 for 6 hours as in D and E. Statistical significance was determined by one-way ANOVA and Tukey multiple comparison test (F, n = 30 cells and G, N = 5, **p<0.01). (H) Generation of MDA-MB-231 cells stably expressing mCherry-FilGAP resistant to *FilGAP* siRNA KD#1 (see Materials and Methods). Expression of endogenous FilGAP and mCherry-FilGAP KD^r^ after *FilGAP* siRNA treatment was confirmed by immunoblotting using an anti-FilGAP antibody. (I) and (J) Quantification of the cells shown in H as in D and E. Statistical significance was determined as in F and G (I, n = 30 cells and J, N = 5, *p<0.05, **p<0.01).

**Fig. 3:**
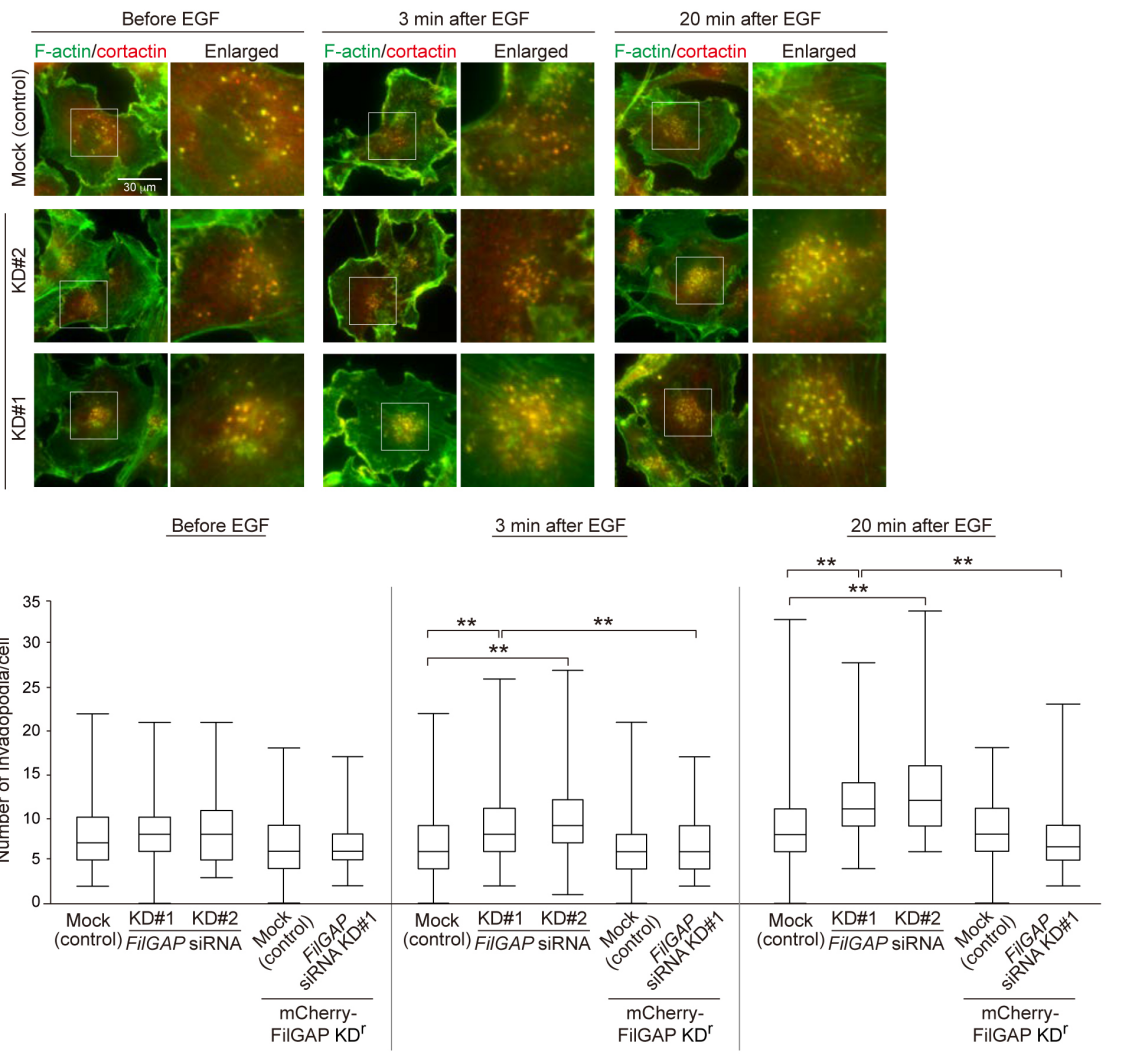
Depletion of FilGAP enhances invadopodia emergence in MDA-MB-231 cells stimulated with EGF (A) Images of invadopodia in control and FilGAP-depleted MDA-MB-231 cells after EGF stimulation. MDA-MB-231 cells were cultured on the gelatin for 1 hour and serum-starved for 3 hours. The serum-starved cells were fixed after the treatment with EGF for the indicated time (3 and 20 min) and stained with an anti-cortactin antibody and phalloidin for F-actin. Enlarged images of the boxed regions are shown. (B) Quantification of the number of invadopodia per cell of the cells shown in A (see Materials and Methods). Statistical significance was determined by one-way ANOVA and Tukey multiple comparison test (n = 50 cells, **p<0.01).

Immunofluorescence was performed as previously described (Saito *et al.*, 2021). In the cases described, cells were fixed after treatment with cytoskeletal buffer (CSK buffer) containing 0.5% Triton X-100 for 2 min to clearly visualize cytoskeletal structures, as previously performed (Morishita *et al.*, 2015). Cells were observed under an Olympus IX73 fluorescence microscope (Olympus, Tokyo, Japan) equipped with a charged-couple device (CCD) camera (ORCA-flash 2.8; Hamamatsu photonics, Hamamatsu, Japan) as previously described (Saito *et al.*, 2021). For the acquirement of confocal images, cells were observed under an Olympus IX81 fluorescence microscope equipped with an electron-multiplying charge-coupled device camera (iXon Ultra, Andor Technology, Tokyo, Japan) as previously described (Hatakeyama *et al.*, 2019). The super-resolution images were also acquired by a laser scanning confocal microscope (LSM880 with Airyscan 2) on an inverted Axio Observer (Carl Zeiss, Jena Germany) through a Plan-Apochromat 63x/1.4 Oil DIC M27 objective (Carl Zeiss). All images were analyzed by ImageJ (National Institutes of Health, NIH, Bethesda, MD, USA). ECM degradation was visualized as darker areas on the gelatin due to proteolytic removal of the fluorescent gelatin (Oregon Green 488-gelatin). In Fig. 1C, E, H, and K, ECM degradation efficiency was calculated as the darker areas divided by the cell areas (visualized by F-actin staining) in 10–20 fields selected randomly for each experiment, as previously described (Martin *et al.*, 2012). In Fig. 2D, F, and I, invadopodia with ECM degradation were analyzed by counting dots of cortactin in the gelatin-degraded spots of a cell. In Fig. 2E, G, and J, the number of cells with invadopodia was counted in 10–20 fields selected randomly for each experiment. In Fig. 3B, to analyze invadopodia in a cell after EGF stimulation, cells were cultured on gelatin for 1 hour and serum-starved for 3 hours. The serum-starved cells were fixed after the treatment with 50 ng/ml EGF for the indicated time and stained with an anti-cortactin antibody and phalloidin for F-actin. Invadopodia after EGF stimulation was analyzed by counting co-localized cortactin and F-actin dots. In Fig. 5, to analyze the localization of FilGAP and PI(3,4)P_2_ at invadopodia in cells on gelatin, the super-resolution images were acquired by LSM880 with Airyscan 2. Immunofluorescence images of cortactin were first acquired and the grayscale images were converted to binary images by the “Threshold” command of ImageJ. A dot size was measured by the “Analyze particles” command of ImageJ and particles (0.05–0.8 μm diameter) were counted as invadopodia. Immunofluorescent dots of HA-FilGAP constructs (R175A and R39C) or PI(3,4)P_2_ probe (GFP-PLEKHA1) in cortactin dots analyzed as above were counted as localized to invadopodia. The localization to invadopodia was calculated as the number of FilGAP or PI(3,4)P_2_ dots in cortactin dots (invadopodia) divided by the number of invadopodia in a cell (Fig. 5C, F, G, I, and J). In Fig. 5D, co-localization of FilGAP and PI(3,4)P_2_ was calculated as the number of FilGAP dots in invadopodia divided by the number of PI(3,4)P_2_ dots in invadopodia.

### Time-lapse microscopy

**Fig. 4:**
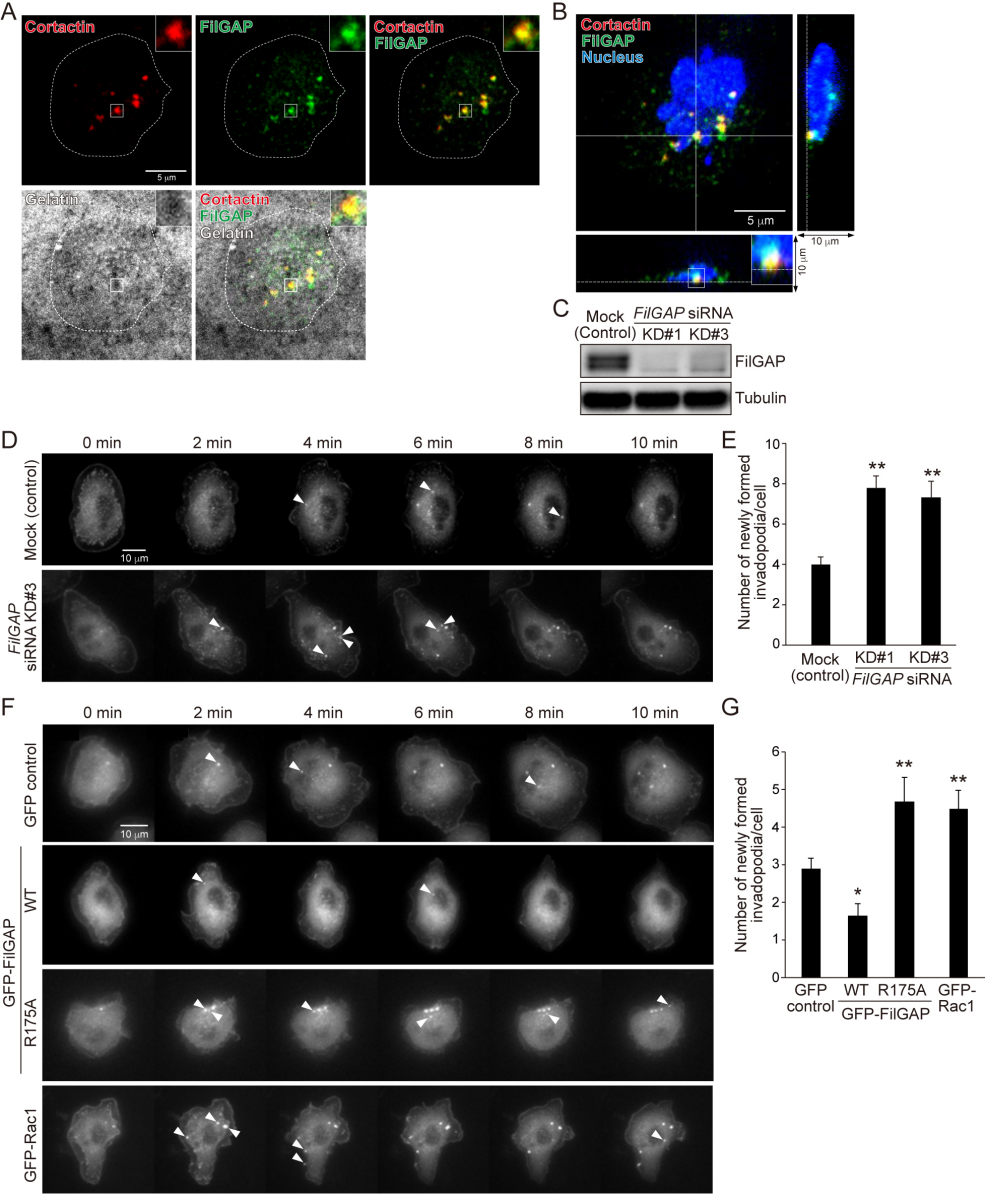
FilGAP suppresses invadopodia emergence by inactivating Rac1 in MTLn3 cells stimulated with EGF (A) Localization of FilGAP at the invadopodia in MTLn3 cells on the gelatin. The cell culture and immunofluorescence were as in Fig. 2A. Dotted lines show cell outlines. (B) Confocal z-stack image of the cell shown in A. The image view was as in Fig. 2B. (C) Immunoblot showing that FilGAP was depleted by *FilGAP* siRNAs in MTLn3 cells. (D) Time-lapse images of control and FilGAP-depleted MTLn3 cells after EGF stimulation. MTLn3 cells expressing mCherry-cortactin were cultured as in Fig. 3. Images of mCherry-cortactin were acquired at 2-min intervals 1 hour after EGF stimulation and the images of the first 10 min were shown (see Materials and Methods). Arrowheads indicate newly emerging spots of mCherry-cortactin in a cell after EGF stimulation. (E) Quantification of the number of newly formed invadopodia per cell of the cells shown in D (see Materials and Methods). Statistical significance was determined by one-way ANOVA and Dunnett multiple comparison test (vs mock cells, n≥30 cells, **p<0.01). (F) Time-lapse images of MTLn3 cells co-transfected with mCherry-cortactin and GFP (control), GFP-FilGAP constructs (WT and R175A), or GFP-Rac1 after EGF stimulation. Images of mCherry-cortactin were as in D. (G) Quantification of the cells shown in F as in E. Statistical significance was determined as in E (vs GFP control cells, n≥30 cells, *p<0.05, **p<0.01).

**Fig. 5:**
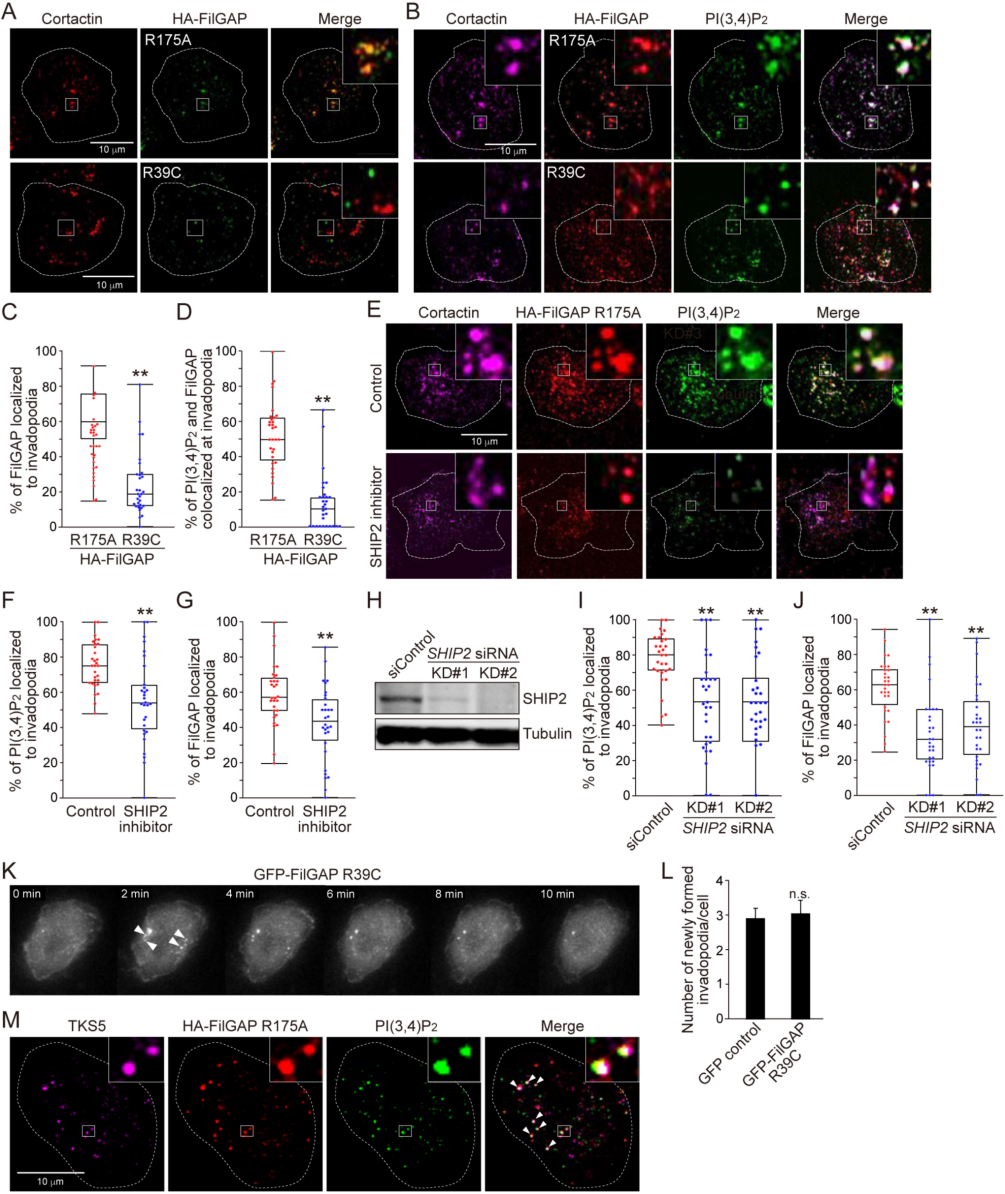
FilGAP localizes to invadopodia through its PH domain binding to PI(3,4)P_2_ in MTLn3 cells (A) Localization of HA-FilGAP constructs (R175A and R39C) at the invadopodia in MTLn3 cells on the gelatin. The cell culture of HA-FilGAP-transfected MTLn3 cells was as in Fig. 2A. After the culture, the cells were fixed after CSK treatment and stained with anti-HA and anti-cortactin antibodies, and the super-resolution images were acquired. Dotted lines show cell outlines and insets show enlarged images of the boxed regions. (B) Co-localization of HA-FilGAP constructs (R175A and R39C) and PI(3,4)P_2_ at the invadopodia in MTLn3 cells on the gelatin. The cell culture of MTLn3 cells co-transfected with HA-FilGAP constructs and GFP-PLEKHA1 (PI[3,4]P_2_ probe) was as in Fig. 2A. After the culture, the cells were fixed after CSK treatment and stained with anti-HA, anti-GFP, and anti-cortactin antibodies. Microscopic observation was as in A. (C) Quantification of FilGAP localized to invadopodia of the cells shown in B (see Materials and Methods). Statistical significance was determined by Student’s *t*-test (n = 30 cells, **p<0.01). (D) Quantification of the co-localization of PI(3,4)P_2_ and FilGAP at the invadopodia of the cells shown in B (see Materials and Methods). Statistical significance was determined as in C (n = 30 cells, **p<0.01). (E) Localization of HA-FilGAP R175A at the invadopodia in MTLn3 cells treated with SHIP2 inhibitor on the gelatin. The cell culture, immunofluorescence, and microscopic observation were as in B. The cells seeded on the gelatin were treated with 10 μM SHIP2 inhibitor (AS1949490) for 6 hours. (F) and (G) Quantification of PI(3,4)P_2_ (F) and FilGAP (G) localized to invadopodia of the cells shown in E as in C. Statistical significance was determined as in C (n = 30 cells, **p<0.01). (H) Immunoblot showing that SHIP2 was depleted by *SHIP2* siRNAs in MTLn3 cells. SHIP2 was detected by immunoblotting using an anti-SHIP2 antibody. (I) and (J) Quantification of PI(3,4)P_2_ (I) and FilGAP (J) localized to invadopodia of the cells shown in H as in C. Statistical significance was determined by one-way ANOVA and Dunnett multiple comparison test (vs siControl cells, n = 30 cells, **p<0.01). (K) Time-lapse images of MTLn3 cells co-transfected with mCherry-cortactin and GFP-FilGAP R39C after EGF stimulation. Images of mCherry-cortactin were as in Fig. 4D. (L) Quantification of the cells shown in K as in Fig. 4E. GFP control shows the data in Fig. 4G. Statistical significance was determined by Student’s *t*-test (n≥30 cells, n.s. = not significant). (M) Co-localization of TKS5 and HA-FilGAP R175A in MTLn3 cells on the gelatin. The cell culture, immunofluorescence, and microscopic observation were as in B. TKS5 was visualized using an anti-TKS5 antibody. Arrowheads in the merged image indicate the co-localization of TKS5 and FilGAP at PI(3,4)P_2_-localized sites. Enlarged images of the boxed regions were representative images of the co-localization.

MTLn3 cells were co-transfected with mCherry-cortactin and GFP (control), GFP-FilGAP constructs (WT, R175A, and R39C), or GFP-Rac1 for 24 hours. The transfected cells were seeded on gelatin and EGF stimulation was performed as described above. The EGF-stimulated cells were immediately examined under an Olympus IX81 fluorescence microscope with a 100× objective lens (Olympus), and an EMCCD camera (iXon3; Andor). Images were acquired at 37°C every 2 min for 1 hour and analyzed by ImageJ and MetaMorph software (Molecular Devices, San Jose, CA, USA). In Fig. 4D, F, and Fig. 5K, newly emerging spots of mCherry-cortactin in a cell 1 hour after EGF stimulation were counted as newly formed invadopodia.

### Statistical analysis

The statistical significance was accessed by a two-tailed unpaired Student’s *t*-test. The statistical significance was also performed using one-way ANOVA followed by Dunnett or Tukey multiple comparison tests. Differences were considered to be statistically significant at a *P* value of <0.05. Error bars (Standard error of the mean, S.E.M.) and *P* values were determined from the results of at least three experiments.

## Results

### FilGAP inhibits ECM degradation of MDA-MB-231 cells

To first elucidate the role of FilGAP in the ECM degradation of tumor cells, we performed the degradation assay of gelatin matrices using highly invasive MDA-MB-231 human breast carcinoma cells. MDA-MB-231 cells degrade gelatin with high efficiency and are therefore suitable to analyze for the degradation assay. Two independent siRNAs targeting human *FilGAP* (KD#1 and KD#2) reduced the expression of endogenous FilGAP in MDA-MB-231 cells (Fig. 1A). Depletion of FilGAP resulted in a significant increase in gelatin degradation (Fig. 1B and C). This increase in gelatin degradation by FilGAP depletion was reduced by the treatment of NCS23766, which is an effective Rac1 inhibitor for MDA-MB-231 cells (Saito *et al.*, 2012; 2021) (Fig. 1D and E). We next generated MDA-MB-231 cells stably overexpressing FilGAP (Fig. 1F) and found that forced expression of FilGAP significantly decreases the gelatin degradation (Fig. 1G and H). We furthermore transfected the stable cell lines with *FilGAP* siRNAs (Fig. 1I). As a result, FilGAP depletion increased the degradation in both control and FilGAP-overexpressing stable cells (Fig. 1J and K). The degradation efficiency seemed to be dependent on the expression level of FilGAP (Fig. 1I). These results show that FilGAP inhibits ECM degradation in breast cancer cells, probably by inactivating Rac1.

### Depletion of FilGAP promotes invadopodia formation in MDA-MB-231 cells

The effect of FilGAP on the ECM degradation of MDA-MB-231 cells (Fig. 1) suggests that FilGAP regulates the formation of invadopodia in breast cancer cells. Therefore, we next analyzed invadopodia formation using MDA-MB-231 cells. We first investigated whether endogenous FilGAP localizes at the invadopodia in MDA-MB-231 cells on the gelatin using an anti-FilGAP antibody (Ohta *et al.*, 2006; Saito *et al.*, 2021). Endogenous FilGAP co-localized with cortactin, a core component of invadopodia, at the spot of gelatin degradation in MDA-MB-231 cells (Fig. 2A) and the co-localization was observed at the ventral surface of the cells (Fig. 2B), indicating the localization of FilGAP to invadopodia. To examine the role of FilGAP in invadopodia of MDA-MB-231 cells, we analyzed dots of cortactin in the gelatin-degraded spots of a cell as invadopodia (Fig. 2C), which are at a mature stage promoting ECM degradation by matrix metalloproteases (Artym *et al.*, 2006). Depletion of FilGAP in MDA-MB-231 cells resulted in a significant increase in the number of ECM-degrading invadopodia per cell (Fig. 2C and D) and the number of cells with the invadopodia (Fig. 2E). This increase in invadopodia formation by FilGAP depletion was reduced by Rac1 inhibition (Fig. 2F and G), as well as that in ECM degradation (Fig. 1D and E). We furthermore generated MDA-MB-231 cells stably expressing mCherry-tagged *FilGAP* siRNA-resistant mutant (mCherry-FilGAP KD^r^) (Fig. 2H). Expression of mCherry-FilGAP KD^r^ reduced ECM-degrading invadopodia formation of FilGAP-depleted cells (Fig. 2I and J). Thus, FilGAP depletion promoted the formation of ECM-degrading invadopodia in MDA-MB-231 cells, suggesting that FilGAP influences mature invadopodia formation in breast cancer cells.

### Depletion of FilGAP enhances invadopodia emergence in MDA-MB-231 cells stimulated with EGF

Treatment of MDA-MB-231 cells with EGF induces the emergence of invadopodia (Moshfegh *et al.*, 2014; Oser *et al.*, 2010). To next analyze EGF-induced invadopodia, MDA-MB-231 cells on the gelatin were serum-starved and stimulated with EGF, and dots co-localized with F-actin and cortactin in the cells 3 or 20 min after EGF stimulation were counted as invadopodia. In MDA-MB-231 cells before EGF stimulation, the number of invadopodia was not changed by the depletion of FilGAP (Fig. 3A and B). When the cells were stimulated with EGF, FilGAP-depleted cells showed a significant increase in the number of invadopodia compared with control cells (Fig. 3A and B). Expression of mCherry-FilGAP KD^r^ reduced EGF-induced invadopodia emergence of FilGAP-depleted cells (Fig. 3A and B). These results suggest that FilGAP regulates the emergence of invadopodia in breast cancer cells.

### FilGAP suppresses invadopodia emergence by inactivating Rac1 in MTLn3 cells stimulated with EGF

We furthermore analyzed the emergence of invadopodia by time-lapse imaging using MTLn3 rat breast carcinoma cells. MTLn3 cells do not degrade the gelatin with high efficiency compared to MDA-MB-231 cells, but cortactin dots (invadopodia) of MTLn3 cells are larger and easier to observe under time-lapse microscopy than those of MDA-MB-231 cells. Therefore, we used MTLn3 cells instead of MDA-MB-231 cells for the time-lapse imaging. We first confirmed the localization of FilGAP in MTLn3 cells on the gelatin and the efficiency of FilGAP depletion by siRNAs targeting human *FilGAP* (KD#1) and rat *FilGAP* (KD#3) in the cells. Endogenous FilGAP co-localized with cortactin at the ventral surface of MTLn3 cells, as well as MDA-MB-231 cells (Fig. 4A and B), and the expression of FilGAP was efficiently reduced by the siRNAs (Fig. 4C). Treatment of MTLn3 cells with EGF, similar to MDA-MB-231 cells, induces invadopodia emergence (Oser *et al.*, 2009; Yamaguchi *et al.*, 2005). MTLn3 cells were transfected with mCherry-cortactin, and newly emerging spots of mCherry-cortactin in a cell 1 hour after EGF stimulation were counted as newly formed invadopodia. Many spots of mCherry-cortactin were observed to appear within 10 min after EGF stimulation in all experimental cells. When FilGAP was depleted by siRNAs, MTLn3 cells showed a significant increase in the number of newly formed invadopodia compared with control cells (Fig. 4D and E, Movies S1–3). Conversely, the forced expression of GFP-tagged FilGAP (WT) decreased it compared with GFP-expressing control cells (Fig. 4F and G, Movies S4 and 5). We next examined whether the effect of FilGAP overexpression is dependent on its GAP activity. As a result, the expression of GFP-GAP-deficient FilGAP mutant (R175A), a dominant negative mutant (Saito *et al.*, 2021), increased the number of newly formed invadopodia, as well as FilGAP depletion (Fig. 4E and F, Movie S6), suggesting that FilGAP inhibits invadopodia emergence by inactivating Rac1. Consistent with this idea, the overexpression of GFP-Rac1 promoted it (Fig. 4F and G, Movie S7). Taken together with the results of Fig. 3, our data suggest that FilGAP inactivates Rac1 and suppresses the emergence of invadopodia in breast cancer cells.

### FilGAP localizes to invadopodia through its PH domain binding to PI(3,4)P_2_ in MTLn3 cells

FilGAP contains a PH domain at its N-terminus and binds phosphatidylinositol 3,4,5-trisphosphate (PIP_3_) and PI(3,4)P_2_ (Kawaguchi *et al.*, 2014). It is known that PI(3,4)P_2_ is distributed on membranes of the invadopodia (Murphy and Courtneidge, 2011; Sharma *et al.*, 2013). In breast cancer cells, PI(3,4)P_2_ is uniformly distributed at the beginning of precursor formation, then accumulates in growing invadopodia, and is enriched at its site with maturation (Sharma *et al.*, 2013). We investigated whether FilGAP localizes to invadopodia in a PI(3,4)P_2_-dependent manner. For the localization analysis, MTLn3 cells were used because invadopodia of the cells are large and easy to quantify them as described above. we first transfected MTLn3 cells with HA-tagged FilGAP R39C mutant, which is unable to bind PIP_3_ and PI(3,4)P_2_ (Kawaguchi *et al.*, 2014), and examined its localization in the cells on the gelatin. Because the transfection of HA-FilGAP WT decreased the number of cortactin dots (invadopodia), its localization analysis and quantification appeared to be difficult. Therefore, we compared the localization of HA-FilGAP R175A as a control to that of the R39C mutant. HA-FilGAP R175A as well as endogenous FilGAP (Fig. 4A) co-localized with cortactin in MTLn3 cells and showed a high frequency of co-localization (Fig. 5A and C). On the other hand, the co-localization of HA-FilGAP R39C mutant was significantly low compared with HA-FilGAP R175A (Fig. 5A and C). We also examined the co-localization of FilGAP and PI(3,4)P_2_ on membranes in MTLn3 cells on the gelatin using a PI(3,4)P_2_-specific probe, GFP-tagged PLEKHA1 (Ivetac *et al.*, 2005). As a result, FilGAP R175A co-localized with PI(3,4)P_2_ at a higher frequency in invadopodia than the R39C mutant (Fig. 5B and D). SH2-containing inositol 5'-phosphatase 2 (SHIP2) localizes at the invadopodia in breast cancer cells and regulates PI(3,4)P_2_ levels locally at its sites by dephosphorylating PIP_3_ (Sharma *et al.*, 2013). We next treated MTLn3 cells with a SHIP2 inhibitor AS1949490, which reduces the amount of PI(3,4)P_2_ in the whole cell and at the invadopodia in MTLn3 cells (Sharma *et al.*, 2013), and examined the FilGAP localization at the invadopodia of the cells on the gelatin. The SHIP2 inhibitor treatment significantly decreased the localization of both PI(3,4)P_2_ and FilGAP to invadopodia (Fig. 5E–G). In addition, we performed the depletion of SHIP2 by siRNAs. Two independent siRNAs targeting rat *SHIP2* (KD#1 and KD#2) reduced the expression of endogenous SHIP2 in MTLn3 cells (Fig. 5H), and SHIP2 depletion resulted in a significant decrease in PI(3,4)P_2_ and FilGAP localization to invadopodia (Fig. 5I and J). These results suggest that FilGAP localizes to the invadopodia in a PI(3,4)P_2_-, but probably not PIP_3_-dependent manner. Finally, we performed an EGF-stimulation experiment using a GFP-FilGAP R39C mutant by time-lapse microscopy. Forced expression of the R39C mutant did not decrease the number of newly formed invadopodia as GFP control did (Fig. 5K and L, Movie S8). These results suggested that the recognition of PI(3,4)P_2_ by the PH domain of FilGAP was essential for the FilGAP function to downregulate invadopodia emergence.

## Discussion

**Fig. 6:**
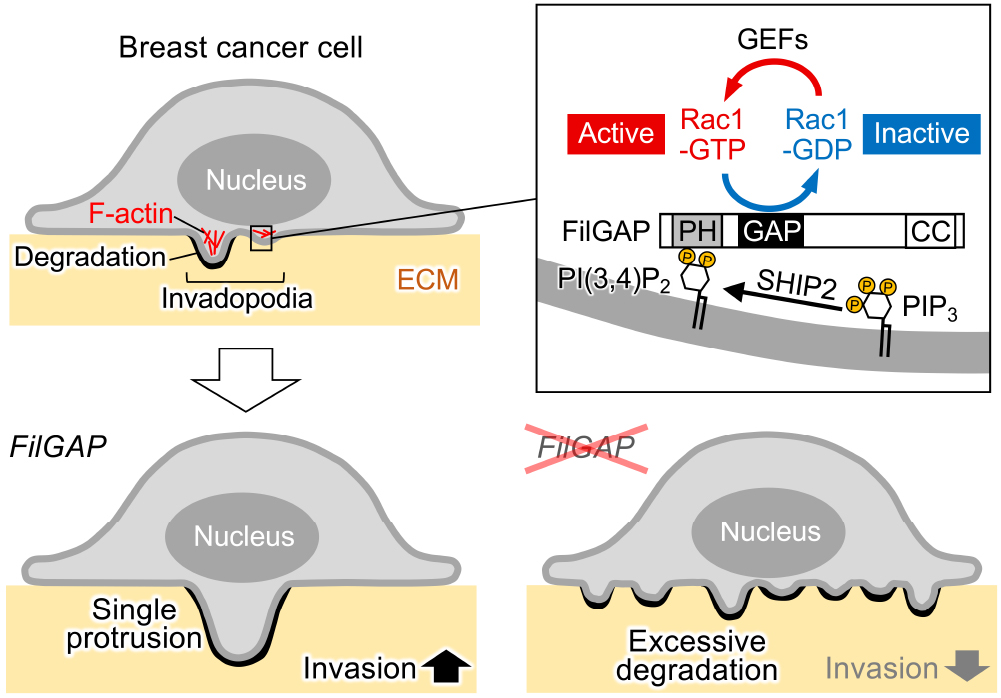
Schematic model diagram of breast cancer invasion into the ECM regulated by FilGAP FilGAP binds PI(3,4)P_2_ on membranes through its PH domain and localizes to invadopodia, leading to the inhibition of invadopodia formation by inactivating Rac1. FilGAP may inhibit multiple invadopodia formations and excessive ECM degradation throughout the cell and results in the formation of a single protrusion for effective breast cancer invasion.

Our findings show that FilGAP regulates invadopodia formation in breast cancer cells. In MDA-MB-231 cells, the depletion of FilGAP promoted ECM degradation and its forced expression decreased the degradation, indicating that FilGAP inhibits ECM degradation in breast cancer cells (Fig. 1). The depletion of FilGAP promoted the formation of invadopodia with ECM degradation in MDA-MB-231 cells (Fig. 2). It is likely that FilGAP regulates invadopodia formation and consequently affects ECM degradation. Furthermore, EGF-stimulation experiments using MDA-MB-231 and MTLn3 cells showed that FilGAP suppresses the emergence of invadopodia by inactivating Rac1 (Fig. 3 and 4). We finally investigated the mechanism of FilGAP localization using MTLn3 cells and found that FilGAP binds PI(3,4)P_2_ on membranes through its PH domain and localizes to invadopodia (Fig. 5 and 6). Thus, we identified FilGAP as a negative regulator of invadopodia formation in breast cancer cells.

Rho small GTPases govern the formation of invadopodia by regulating actin dynamics at each process (Rivier *et al.*, 2021). Regarding Rac1, its roles appear to be cell- and context-dependent. Rac1 activity is required for mature invadopodia formation and/or ECM degradation in some cancer cell lines, while Rac1 regulates invadopodia disassembly at the final stage in others (Goicoechea *et al.*, 2017; Moshfegh *et al.*, 2014; Nakahara *et al.*, 2003; Nascimento *et al.*, 2011; Pignatelli *et al.*, 2012). In any case, Rac1 is suggested to be involved in the late process. The details of whether Rac1 participates in the emergence of invadopodia at the early stage are still unclear (Rivier *et al.*, 2021), but our time-lapse imaging data may suggest that Rac1 mediates invadopodia emergence and FilGAP inactivates Rac1 at this stage, resulting in suppression of invadopodia formation in breast cancer cells. On the other hand, our results do not exclude the possibility that FilGAP plays a role in the regulation of invadopodia at the mature stage. It will be interesting to determine how the function of FilGAP is regulated at each stage of invadopodia assembly and maturation.

The production of PI(3,4)P_2_ through SHIP2 is important for the progression of invadopodia (Murphy and Courtneidge, 2011; Sharma *et al.*, 2013; Yamaguchi, 2012). Increased PI(3,4)P_2_ production at the early stage recruits major components whose PH domains bind selectively to PI(3,4)P_2_, including tyrosine kinase substrate with five SH3 domains (TKS5) and lamellipodin, and supports growing invadopodia, leading to its maturation and ECM degradation (Krause *et al.*, 2004; Sharma *et al.*, 2013). Here we reported FilGAP as a novel protein that binds PI(3,4)P_2_ at invadopodia. As above, FilGAP negatively regulates invadopodia formation, while TKS5 and lamellipodin promote it. We examined whether FilGAP (HA-FilGAP R175A and endogenous FilGAP) colocalizes or competes with TKS5 at PI(3,4)P_2_-localized sites in MTLn3 cells on the gelatin, and found that FilGAP is capable of adequate co-localization with TKS5 at its sites (Fig. 5M and data not shown). This result suggests that FilGAP does not interfere with TKS5 recruitment to invadopodia by sequestering PI(3,4)P_2_. Consistent with this idea, forced expression of HA-FilGAP R175A localized to invadopodia dominant-negatively promoted the emergence of invadopodia (Fig. 4). The bindings of these distinct proteins to PI(3,4)P_2_ could be reversible and result in the dynamic behavior of invadopodia in breast cancer cells, as previously proposed (Sharma *et al.*, 2013). Further studies will be necessary to determine the mechanism of how the PI(3,4)P_2_ binding of these proteins to each other is spatiotemporally regulated at invadopodia. In addition to PI(3,4)P_2_, FilGAP has the ability to bind PIP_3_ through its PH domain (Kawaguchi *et al.*, 2014). While we previously reported that FilGAP localizes to the cell front through its binding to PIP_3_ and regulates tumor cell migration in the ECM (Saito *et al.*, 2021), we here showed the importance of FilGAP binding to PI(3,4)P_2_ for its localization to invadopodia. These findings suggest the selective binding of FilGAP to PIs at each site of cellular events. Phosphoinositide 3-kinase (PI3K) produces PIP_3_, and PIP_3_ is dephosphorylated by SHIP2 to form PI(3,4)P_2_ at invadopodia (Sharma *et al.*, 2013; Yamaguchi, 2012). The production of PIP_3_ through PI3K is also responsible for the regulation of invadopodia (Khalil *et al.*, 2016; Yamaguchi *et al.*, 2011). Our results do not exclude the possibility that FilGAP binds PIP_3_ at invadopodia. Therefore, there may be an intriguing mechanism that FilGAP transiently binds PIP_3_ at invadopodia and its function is regulated.

We previously reported that FilGAP promotes breast cancer invasion into the ECM *in vitro* and *in vivo* (Saito *et al.*, 2021; 2012). In addition, FilGAP controls the front-rear polarity of invading breast cancer cells and is required for maintaining effective cell migration in the ECM (Saito *et al.*, 2021). On the other hand, we here showed that in breast cancer cells, FilGAP suppresses invadopodia formation, which is the first migratory process for cancer invasion. Why does FilGAP promote cancer invasion even though it suppresses invadopodia formation? Invadopodia are transmitted to a single and large protrusion for cell invasion into the ECM (Linder *et al.*, 2011; Schoumacher *et al.*, 2010). One possibility is that FilGAP inhibits its multiple formations and excessive ECM degradation throughout the cell and results in the formation of a single protrusion for effective cell invasion (Fig. 6). In fact, cells with many small invadopodia can not form a single protrusion and invade into the ECM (Hagedorn *et al.*, 2013; Lohmer *et al.*, 2014). All together with this study and our previous work (Saito *et al.*, 2021; 2012), we propose that FilGAP inactivates Rac1 precisely at each step of cell invasion and contributes to the progression of breast cancer cells.

Studies on Rho GAPs in cancer progression including invadopodia are increasing (Kreider-Letterman *et al.*, 2022). Recently, ARHGAP17, a Cdc42-specific GAP, was identified as a negative regulator of invadopodia in breast cancer cells by shRNA screening for Rho GAPs (Kreider-Letterman *et al.*, 2023). We here showed that FilGAP similar to ARHGAP17 negatively regulates invadopodia formation by inactivating Rac1. Because there are multiple Rho GAPs for each Rho GTPase, with more than 60 Rho GAPs encoded in the human genome (Tcherkezian and Lamarche-Vane, 2007), various Rho GAPs besides the above proteins could have redundant functions in invadopodia formation. For example, SH3BP1 and/or ARHGAP44, two closely related members of ARHGAP17, may be able to compensate for the loss of ARHGAP17 (Kreider-Letterman *et al.*, 2023). FilGAP also has two subfamily members, ARHGAP22 and ARHGAP25, all sharing a similar domain structure and specificity for Rac1 (Nakamura, 2013; Ohta *et al.*, 2006). These proteins are involved in cell invasion of carcinoma, melanoma, or sarcoma cells (Sanz-Moreno *et al.*, 2008; Tao *et al.*, 2019; Thuault *et al.*, 2016; Xu *et al.*, 2019). It will be interesting to examine the roles of ARHGAP22 and ARHGAP25 in invadopodia formation of tumor cells. Future studies should explore how individual Rho GAPs regulate Rho small GTPase activities to regulate invadopodia dynamics for cancer invasion.

## Author Contributions

KS, SO, YC, RT, and RO conducted experiments and analyzed data; KM and TT supported super-resolution microscopic observations. HH supported confocal microscopic observations; KS and YO designed the research and wrote the manuscript. All authors reviewed the manuscript and provided approval for submission.

## Conflict of Interest

The authors have no conflicts of interest to disclose.

## Supplementary Data

Supplemental movies are available in the Cell Structure and Function online.

## Abbreviations

CSK: cytoskeletal buffer
ECM: extracellular matrix
EGF: epidermal growth factor
F-actin: filamentous actin
GAP: GTPase-activating protein
GEF: guanine nucleotide exchange factor
PBS: phosphate-buffered saline
PCR: polymerase chain reaction
PMSF: phenylmethylsulfonyl fluoride
PI3K: phosphoinositide 3-kinase
PI(3,4)P_2_: phosphatidylinositol 3,4-bisphosphate
PIP_3_: phosphatidylinositol 3,4,5-trisphosphate
PH: pleckstrin-homology
SDS: sodium dodecyl sulfate
SHIP2: SH2-containing inositol 5'-phosphatase 2
siRNA: small interfering RNA
TKS5: tyrosine kinase substrate with five SH3 domains

## Supplementary Material

Movie S1 Time-lapse movie of Mock (control) MTLn3 cells, related to Fig. 4D.

Movie S2 Time-lapse movie of FilGAP-depleted (KD#1) MTLn3 cells, related to Fig. 4D.

Movie S3 Time-lapse movie of FilGAP-depleted (KD#3) MTLn3 cells, related to Fig. 4D.

Movie S4 Time-lapse movie of GFP control MTLn3 cells, related to Fig. 4F.

Movie S5 Time-lapse movie of GFP-FilGAP WT-overexpressing MTLn3 cells, related to Fig. 4F.

Movie S6 Time-lapse movie of GFP-FilGAP R175A-overexpressing MTLn3 cells, related to Fig. 4F.

Movie S7 Time-lapse movie of GFP-Rac1-overexpressing MTLn3 cells, related to Fig. 4F.

Movie S8 Time-lapse movie of GFP-FilGAP R39C-overexpressing MTLn3 cells, related to Fig. 5K.
